# X-inactivation informs variance-based testing for X-linked association of a quantitative trait

**DOI:** 10.1186/s12864-015-1463-y

**Published:** 2015-03-25

**Authors:** Li Ma, Gabriel Hoffman, Alon Keinan

**Affiliations:** Department of Animal and Avian Sciences, University of Maryland, College Park, MD 20740 USA; Department of Biological Statistics and Computational Biology, Cornell University, Ithaca, NY 14850 USA; Present address: Icahn Institute for Genomics and Multiscale Biology, Department of Genetics and Genomic Sciences, Icahn School of Medicine at Mount Sinai, New York, USA

## Abstract

**Background:**

The X chromosome plays an important role in human diseases and traits. However, few X-linked associations have been reported in genome-wide association studies, partly due to analytical complications and low statistical power.

**Results:**

In this study, we propose tests of X-linked association that capitalize on variance heterogeneity caused by various factors, predominantly the process of X-inactivation. In the presence of X-inactivation, the expression of one copy of the chromosome is randomly silenced. Due to the consequent elevated randomness of expressed variants, females that are heterozygotes for a quantitative trait locus might exhibit higher phenotypic variance for that trait. We propose three tests that build on this phenomenon: 1) A test for inflated variance in heterozygous females; 2) A weighted association test; and 3) A combined test. Test *1* captures the novel signal proposed herein by directly testing for higher phenotypic variance of heterozygous than homozygous females. As a test of variance it is generally less powerful than standard tests of association that consider means, which is supported by extensive simulations. Test *2* is similar to a standard association test in considering the phenotypic mean, but differs by accounting for (rather than testing) the variance heterogeneity. As expected in light of X-inactivation, this test is slightly more powerful than a standard association test. Finally, test *3* further improves power by combining the results of the first two tests. We applied the these tests to the ARIC cohort data and identified a novel X-linked association near gene *AFF2* with blood pressure, which was not significant based on standard association testing of mean blood pressure.

**Conclusions:**

Variance-based tests examine overdispersion, thereby providing a complementary type of signal to a standard association test. Our results point to the potential to improve power of detecting X-linked associations in the presence of variance heterogeneity.

## Background

The X chromosome (ChrX) plays a role in complex human disease and quantitative traits [1-4]. Sex-specific differences in prevalence, age of onset and severity have been reported in many human diseases, including cardiovascular diseases, asthma, and autoimmune diseases, as well as a few birth defects, neurological and psychiatric disorders, and some common cancers [4-9]. While many X-linked genes undergo X-inactivation, some degree of expression heterogeneity among females has been reported: 15% of X-linked genes escape inactivation and 10% of X-linked genes exhibit variable patterns of inactivation, which might help explain the widespread gender disparity in disease risk and pathogenesis [3]. As many genome wide association studies (GWAS), however, disregarded or ineffectively analyzed ChrX [10,11], its function in complex diseases and traits remains vague at best. A prerequisite for the development and application of powerful ChrX-wide association studies (XWAS) is a coherent understanding of the problems that have hindered such studies [12-17]. ChrX’s mode of inheritance entails different phenotypic consequences of X-linked polymorphisms, including the exposure of recessive mutations in hemizygous males, a higher chance of dominant mutations affecting females, and more complex mutation models [14,15]. These, in turn, lead to many differences between ChrX and the autosomes that should be carefully accounted for in extending GWAS of the autosomes to efficient XWAS [14].

Why did many GWAS ignore ChrX? Why have studies that took on the challenge of analyzing it rarely found significant associations? Differences between ChrX and the autosomes require special attention lest they lead to reduced statistical power and fewer associations, or—in some cases—even to false positives [17,18]. Among many others, these differences reside in allelic sample size, SNP density on arrays, sex-specific effect sizes, X-inactivation, gene-gene interactions, ascertainment biases, population stratification, and quality control. In addition to many studies discarding ChrX completely due to such analytical complications, some studies initially attempted analysis of ChrX but eventually excluded it after having obtained inflated results, indicative of false positives [personal communications]. A recent meta-analysis that identified 95 loci associated with lipid levels reported four of these exhibited striking sex-specific patterns, while seven additional loci showed a significant association in one sex but not in the sex-combined analysis [19]. Like most studies, it excluded data from ChrX, which we hypothesize is even more likely to harbor such loci with sex-specific association patterns. Another problem of ChrX is introduced by differential ascertainment biases of X-linked variants, which we have shown to plague not only genotyping arrays [20,21], but also next-generation sequencing platforms [22], as well as genotyping arrays designed based on variants discovered from the 1000 Genomes Project [22,23].

Here, we focus on one crucial feature of ChrX that should be considered in association studies, namely dosage compensation and X-inactivation [3,24-28]. X-inactivation was discovered over fifty years ago [29], but it is still unclear whether and how X-inactivation is associated with human complex traits. Most GWAS studies of X-linked variants either ignored X-inactivation [12,30] or addressed it by simply changing the male genotype coding from 0/1 to 0/2, i.e. considering hemizygous males as equivalent to female homozygotes [13,28]. Tailored modeling and testing of X-linked variants is needed to properly incorporate X-inactivation. Wang et. al. recently proposed a likelihood-based test of X-linked association by considering three possible states of X-inactivation—random X-inactivation, skewed X-inactivation, and escape from X-inactivation—and applying three respective association tests that have better power in each scenario [16]. These studies addressed the challenge that X-inactivation poses for association testing. Here, we consider this problem as an opportunity in disguise, as the presence of X-inactivation predicts unique patterns that can be incorporated into association testing. In the presence of X-inactivation female heterozygotes are expected to exhibit elevated stochasticity of expressed variants. For a variant affecting a quantitative trait, this can translate to higher variance in the trait in heterozygous than homozygous females. Several other factors can also lead to different variances: A recent study suggested that gene-gene interactions (epistasis) may cause increased variance in heterozygotes [31], and more generally a mutation can directly disturb the homeostasis of the level of expression of a gene, thus changing the phenotypic variances between genotypic classes of the quantitative trait locus (QTL) [32,33].

While the standard association test considers genetic effect on phenotypic means, a test of genetic effect on phenotypic variance has been developed and applied to detect genetic variants which affect gene expression levels [34,35] and quantitative traits [33,36]. In this study, we extend the test of variance and standard association test to ChrX and tailor them to leverage the observation that heterozygous females are expected to exhibit different variance than homozygous females due to X-inactivation and other factors. We evaluated the performance of the tests of X-linked associations proposed herein by extensive simulations and report scenarios in which they facilitate improved power. Finally, we applied the proposed tests to associate X-linked quantitative trait loci in data from the Atherosclerosis Risk in Communities (ARIC) cohort and report one novel association which was missed by the standard association test.

## Methods

### A simple illustration of X-inactivation and other factors increasing phenotypic variation of heterozygous females

For an X-linked variant affecting a quantitative trait, random X-inactivation can translate to higher variance in the trait in heterozygous females compared to homozygous females. For illustration, consider a simple scenario of one X-linked QTL with two alleles, *Q* and *q*, then the phenotypic model will be,

$$ {y}_i=\mu +{g}_i+{e}_i, $$

where *y*_*i*_ is the phenotype of individual *i*, *μ* is the population mean, *g*_*i*_ is the genotypic value of the QTL for individual *i*, and *e*_*i*_ is a random error of individual *i*, with *Var*(*e*_*i*_) = *σ*^2^. First, consider an extreme case of the QTL undergoing a completely random and uniform X-inactivation, i.e. the same allele is inactivated in all cells that contribute to the studied phenotype. This scenario translates into a genotypic value of females as following:

1 $$ {g}_i=\left\{\begin{array}{c}\hfill 0,\kern2.25em  if\ QTL=qq\hfill \\ {}\hfill 0\  or\ a\  with\  equal\  probability\  of\ 0.5,\kern0.5em  if\ QTL=Qq\hfill \\ {}\hfill a,\kern2.25em  if\ QTL=QQ\hfill \end{array}\right. $$

where *a* is the additive effect of the QTL. For individuals with one of the homozygotes (*QQ* or *qq*) genotype, the phenotypic variation would be just the variance of the random error, *σ*^2^. For individuals with a heterozygous genotype (*Qq*), on average, half of the individuals express the phenotype of *Q* allele and half exhibit the phenotype of *q* allele. Therefore, the phenotypic variance of the heterozygous females will be *a*^2^/4 + *σ*^2^, which is larger than the phenotypic variance of the homozygotes. The level of variance heterogeneity depends on the effect size of the QTL with the difference in variance between heterozygous and homozygous individuals being *a*^2^/4.

This exact equation will not hold in more complex scenarios such as non-uniform inactivation or multiple QTLs. For example, let’s now consider a scenario where the X-inactivation is incomplete: the probability of expressing one QTL allele, *Q*, is 0.75 rather than 0.5. We derived the variance of heterozygous individuals to be 3*a*^2^/16 + *σ*^2^, which is slightly less than the variance of heterozygotes when the inactivation ratio is 0.5. In a general situation of an inactivation ratio of *θ*, the variance of heterozygous individuals is equal to *θ*(1 − *θ*)*a*^2^ + *σ*^2^. When considering multiple X-linked QTLs or multiple tissues with tissue-specific inactivation ratios, the variance heterogeneity will be similar or less pronounced. However, the variance of heterozygous females is expected to be higher in any scenarios of X-inactivation, except for genes that escape X-inactivation.

Besides random X-inactivation, many other factors can also cause differing variances in phenotype across the three genotypic classes. A mutation may affect the homeostasis of the level of expression of a gene, the protein level, or even the level of the final phenotype, thus changing the phenotypic variances across genotypic classes of the QTL [32]. Recent studies have also indicated that genetic interactions may give rise to genotype-dependent variances [31]. Finally, parent-of-origin effect may increase phenotypic variance among those individuals who are heterozygous at the QTL [37].

### A test for X-linked association via inflated variance in heterozygous females

First, we set out to directly test for higher phenotypic variance of heterozygous than homozygous females. Formally, we propose a modified Brown-Forsythe test of equal variances [38]. Suppose *y*_*i|g=j*_ is the phenotypic value of the *i*th individual in the *j*th genotype group (*g*) with *j* = 0, 1, or 2 copies of the reference allele. We first transform the original phenotype to a median-centered phenotypic value by *z*_*i*|*g* = *j*_ = |*y*_*i*|*g* = *j*_ − *ỹ*_*g* = *j*_|, where *ỹ*_*g* = *j*_ is medial *y*_*i|g=j*_ taken over *i*. The null hypothesis is that phenotypic variances of the three genotype groups with *j* = 0, 1, and 2 copies of the reference allele are all equal. The alternative hypothesis is that female heterozygotes have a larger phenotypic variance than others. A test statistic is derived as

$$ {T}_{var}=\frac{\overline{Z_1}-\overline{Z_{0/2}}}{\sqrt{\frac{s_1^2}{n_1}+\frac{s_{0/2}^2}{n_0+{n}_2}}} $$

where $\overline{Z_1}$ is the sample mean of *z*_*i|g=*1_ over *i*, $\overline{Z_{0/2}}$ is the sample mean of *z*_*i|g=*0_ and *z*_*i|g=*2_ combined, ${s}_1^2$ and ${s}_{0/2}^2$ are the sample variances respectively, and *n*_*j*_ is the sample size of *z*_*i|g=j*_. Under the null hypothesis, the statistic follows a *t*-distribution with degrees of freedom given by $df=\frac{{\left({s}_1^2/{n}_1+{s}_{0/2}^2/\left({n}_0+{n}_2\right)\right)}^2}{{\left({s}_1^2/{n}_1\right)}^2/\left({n}_1-1\right)+{\left({s}_{0/2}^2/\left({n}_0+{n}_2\right)\right)}^2/\left({n}_0+{n}_2-1\right)}$. This formulation assumes that female homozygotes for either allele exhibit similar phenotypic variance. However, this assumption can be relaxed with a generalization to an analysis of variance (ANOVA)-based test statistic that allows each of the three genotypes to exhibit different variances. Additionally, this test aims for variable levels of X-inactivation or other contributing factors by simply comparing variances between heterozygous and homozygous females.

### A weighted test for X-linked association that accounts for differential variances

Second, we propose a weighted regression [39] approach for testing X-linked associations to account for the variance inflation caused by factors including X-inactivation. A weighted regression is commonly applied when the residual variance is not constant by assigning less weight to the less precise measurements, and more weight to the more precise measurements. We propose to use the inverse of the empirical variance for each genotypic group as weights, i.e. ${w}_{i\Big|g=j}=1/\widehat{Var}\left({y}_{g=j}\right),$ since the true variances are unknown. Note that *w*_*i|g=j*_ can be different for each of j = 0, 1, or 2 copies of an allele, thereby allowing for different variances between the two female homozygote genotypes and the female heterozygote genotype.

### Combined test of variance and weighted association by Stouffer’s approach

As the two tests described above, the variance-based test and the weighted association test, can capture partially uncorrelated signals and are independent under the null hypothesis, we propose to further improve power of associating X-linked variants by combining the two into a single test statistic using the Stouffer’s Z-score method: p-values of the two tests are transformed to Z scores, *Z*_1_ and *Z*_2_, and the Z-statistic of the combined test would be $\frac{Z_1+{Z}_2}{\sqrt{2}}$. Since the power of the variance test and the weighted association test can be very different, it is desirable to also use a weighted Z-statistic, $\frac{w_1{Z}_1+w{Z}_2}{\sqrt{w_1^2+{w}_2^2}},$ where *w*_1_ and *w*_2_ are the weights of the two tests respectively. In this study, we used equal weights for the two tests, but this can be improved in future studies. Implementation of all three tests developed in this study, including source code, will be made available as part of the next release of our *chromosome X-Wide Analysis tool-Set*, which is freely available for download from http://keinanlab.cb.bscb.cornell.edu/content/tools-data.

### Implementation of standard association testing without variance heterogeneity

For comparison purposes, we also implemented a standard association test in the same way as how ChrX is handled in PLINK [30]. The standard test is similar to the weighted test but assuming equal variances between genotypic groups. Basically, a linear regression model was fitted with females coded as 0, 1, or 2 without considering variance heterogeneity.

### Simulations

Genotype data were simulated under the Hardy-Weinberg Equilibrium (HWE) with given allele frequencies of the QTL. When simulating phenotype data, we considered a null scenario of no association vs. a simple alternative scenario of one X-linked causal variant. Under the null hypothesis, genotype and phenotype data were simulated independently. Under the alternative hypothesis, the genotypic value of an individual was simulated with a complete and uniform X-inactivation process by randomly assigning heterozygous females to express one of the QTL alleles as described in Equation (1), equivalent to female heterozygotes having a genotypic value equal to that of either female homozygotes with equal probability. The phenotypic value was then generated by adding a random error from a standard normal distribution to the genotypic value. To consider scenarios where other factors than X-inactivation contribute to increased variance in heterozygous females, we also simulated increased variance heterogeneity by directly introducing additional random noises to individuals with heterozygous genotypes (10% and 20% of the residual variance). In addition, we varied the sample size from 1000 to 5000, the minor allele frequency of QTL from 0.1 to 0.3, and the effect size of QTL from 0.1 to 0.2. Note that we only included female individuals in our study. For each simulated dataset, we applied the three test statistics as well as a standard association test. To evaluate the Type-I error and power of the proposed tests, for each scenario we repeated the simulation 100,000 times and calculated the type-I error rate and power as the fraction of simulations with a p-value < 0.05 under the null and alternative hypotheses, respectively.

### Application to GWAS data from ARIC

#### Ethics statement

The ARIC study has been approved by the Institutional Review Boards (IRB) of all participating institutions, including the IRB boards of the University of Minnesota, Johns Hopkins University, University of North Carolina, University of Mississippi Medical Center, and Wake Forest University. Because this study analyzed publicly available data, no additional ethical concerns need to be considered beyond those mentioned in the original publications [40].

The Atherosclerosis Risk in Communities (ARIC) Study is a prospective study of atherosclerotic diseases [40]. A total of 15,792 European American and African American individuals were recruited in the baseline examination in 1987–1989, with three triennial follow-up examinations. We included 9,713 European Americans, for whom both phenotype and genotype data were available, in this study. Many atherosclerotic disease related traits were measured in the ARIC study, including total cholesterol (TC), low-density lipoprotein cholesterol (LDL-C), high-density lipoprotein cholesterol (HDL-C), triglyceride (TG), systolic blood pressure (SBP), diastolic blood pressure (DBP), and body mass index (BMI). We obtained ~1 million directly measured SNP genotypes with the Affymetrix 6.0 SNP array and considered 34,527 X-lined SNPs. We applied standard quality control (QC) procedures, including minor allele frequency (>5%), missing rate of SNP (<10%), missing rate of individual (<10%), and Hardy-Weinberg Equilibrium p-value in females (>5 × 10^−5^) [30]. We included a total of 24313 X-linked SNPs after QC, indicating a ChrX-wide significance level of 2 × 10^−6^ after Bonferroni correction. We applied the three proposed tests that incorporate variance heterogeneity as well as the standard association test to the seven quantitative traits from ARIC as described above. In addition, we applied similar QC procedures to the autosomal SNPs and applied the *weighted* and *standard* association tests to the autosomal SNPs after QC.

## Results and discussion

### Evaluation of power and error of tests involving variance heterogeneity

We carried out extensive simulations to evaluate the type-I error and power of the three tests of X-linked association we proposed (Methods), including (1) a test of inflated phenotypic variance in heterozygous females (referred to as *variance* throughout), (2) a weighted test that accounts for differential variance between heterozygous females and homozygous females for each allele (*weighted*), and (3) a combined test of the above two using Stouffer’s Z-score method (*combined*). We also compared this with a standard association test (*standard*). We calculated type-I error rates and power of the four tests as the proportion of simulations with a p-value less than the preselected significance level under the null and alternative hypotheses, respectively. We repeated this for four sample sizes, 1000, 2000, 3000, and 5000. All four tests accurately control for type-I error rate at the desired nominal level of significance (0.05; Table 1).

**Table 1:** **Type-I error rate of the four tests of X-linked associations under various scenarios**

**Sample size**	**Type-I error**			
	**Standard**	**Variance**	**Weighted**	**Combined**
1000	0.0500	0.0492	0.0533	0.0514
2000	0.0504	0.0503	0.0498	0.0503
3000	0.0494	0.0490	0.0505	0.0514
5000	0.0510	0.0486	0.0500	0.0511

Next, we compared the power of the four tests using simulations. We considered two types of scenarios where variance heterogeneity is caused either by random X-inactivation alone or by X-inactivation plus other potential noises affecting heterozygous females (Table 2). In general, the *variance* test of X-inactivation is less powerful than the other association tests, as expected by this test being based on variance, which is generally less powerful than tests of means. However, enhancing the *standard* by accounting for the variance as incorporated in the *variance* test, as accomplished by the *weighted* test, always leads to an increase in power, if only a slight one (Table 2). The performance of the *combined* test largely depends on the power of the *variance* test: it outperforms the *standard* test when the variance test has any power and thereby contributes to the *combined* test statistic. In the set of simulations reported in Table 2, this is only the case in scenarios when an additional source of noise is simulated, especially in cases where the power of the *standard* test is moderate. More generally, the tests that specifically test for variance heterogeneity (*variance* and *combined*) performs much better when faced with higher level of phenotypic noise for heterozygous females as can be observed by comparing the right hand to the left hand of Table 2.

**Table 2:** **Power of the four tests of X-linked associations under various scenarios**

**Simulation parameters**			**X-inactivation in heterozygous females**				**X-inactivation and additional noise in heterozygous females**			
			**Standard**	**Variance**	**Weighted**	**Combined**	**Standard**	**Variance**	**Weighted**	**Combined**
N = 1000	MAF = 0.1	a = 0.1	26.7	5.3	28.8	21.3	26.6	18.0	28.0	33.8
N = 1000	MAF = 0.3	a = 0.1	53.8	6.5	54.2	42.4	52.0	28.9	53.3	63.4
N = 1000	MAF = 0.1	a = 0.2	75.7	7.5	75.9	54.5	73.3	24.4	73.3	72.2
N = 1000	MAF = 0.3	a = 0.2	98.1	11.1	98.2	85.9	97.9	39.0	98.1	96.5
N = 2000	MAF = 0.1	a = 0.1	47.5	5.9	48.2	28.4	45.0	29.3	45.9	56.8
N = 2000	MAF = 0.3	a = 0.1	82.4	6.9	82.4	56.3	81.8	45.4	82.4	87.2
N = 2000	MAF = 0.1	a = 0.2	96.3	10.6	96.3	82.4	94.6	41.1	95.1	95.1
N = 3000	MAF = 0.1	a = 0.1	63.8	5.7	64.0	39.1	62.1	39.5	62.1	73.6
N = 3000	MAF = 0.3	a = 0.1	94.7	7.0	94.7	72.8	93.2	58.8	93.5	96.4
N = 3000	MAF = 0.1	a = 0.2	99.6	12.5	99.6	94.1	99.3	54.3	99.3	99.1
N = 5000	MAF = 0.1	a = 0.1	85.4	6.6	85.5	59.4	83.6	57.5	84.6	92.2

Shown are percentages of simulations where the test in the column positively identifies the QTL with p-value < 0.05. Simulation scenarios include varying sample sizes (N), minor allele frequencies of the QTL (MAF), and effect sizes of the QTL (a).

### The *variance* and *standard* tests captures different association signals

We have shown in Table 2 that the *combined* test outperforms the standard test when the *variance* test has power, indicating the different signals captured by the *variance* and *standard* tests. To evaluate this difference, we conducted a similar set of simulations as described in Table 2, and focused on the results of the *variance* and *standard* tests. To clearly show the difference of the two, we added one scenario with a higher level of variance heterogeneity (20% additional noise in heterozygous individuals). In Table 3, we summarized the fraction of simulations with p-value less than 0.05 for each of the two tests and the fraction of simulations with both tests having a p-value less than 0.05 (shared), with the difference between these fractions measuring the independent signals captured by each test. The expected value of the shared fractions matched the observed shared fractions well (Table 3), thus suggesting the independence of the two tests under the alternative hypothesis. As these two tests capture different signals, when the variance heterogeneity is largely increased without changing the means (in the case of 20% additional noise in heterozygous females), the variance test can possibly outperform the standard test of association (Table 3).

**Table 3:** **Standard and variance tests capture different signals in simulations**

**Simulation parameters**			**X-inactivation and 10% additional noise in heterozygous females**			**X-inactivation and 20% additional noise in heterozygous females**		
			**Standard**	**Variance**	**Shared (expected)**	**Standard**	**Variance**	**Shared (expected)**
N = 1000	MAF = 0.1	a = 0.1	26.5	17.8	4.5 (4.7)	26.9	40.6	10.6 (10.9)
N = 1000	MAF = 0.3	a = 0.1	51.0	29.2	15.1 (14.9)	49.9	62.5	30.8 (31.2)
N = 1000	MAF = 0.1	a = 0.2	73.8	24.0	18.0 (17.7)	73.6	47.5	34.6 (34.9)
N = 1000	MAF = 0.3	a = 0.2	98.1	39.2	37.7 (38.5)	97.8	71.2	63.6 (69.6)
N = 2000	MAF = 0.1	a = 0.1	46.7	29.6	14.2 (13.8)	45.9	65.9	29.8 (30.2)
N = 2000	MAF = 0.3	a = 0.1	81.1	45.2	36.9 (36.7)	80.8	87.2	70.5 (70.5)
N = 2000	MAF = 0.1	a = 0.2	95.7	41.4	39.7 (39.6)	95.2	75.8	72.2 (72.2)
N = 3000	MAF = 0.1	a = 0.1	62.3	39.4	25.4 (24.5)	62.5	82.2	51.5 (51.4)
N = 3000	MAF = 0.3	a = 0.1	93.1	57.9	54.1 (53.9)	93.1	96.0	89.3 (89.4)
N = 3000	MAF = 0.1	a = 0.2	99.3	54.8	53.6 (54.4)	99.2	89.7	88.9 (89.0)
N = 5000	MAF = 0.1	a = 0.1	83.5	57.0	47.6 (47.6)	82.3	95.7	78.7 (78.7)

Shown are percentages of simulations where the test in the column positively identifies the QTL with p-value < 0.05. The shared column denotes the case where both the standard and the variance test significantly identify the QTL, and the differences between the shared and the two tests indicate the different signals the two tests capture. The expected is calculated by assuming the standard and variance tests are independent. Simulation scenarios include varying sample sizes (N), minor allele frequencies of the QTL (MAF), and effect sizes of the QTL (a).

### Application to XWAS in ARIC data

**Figure 1:**
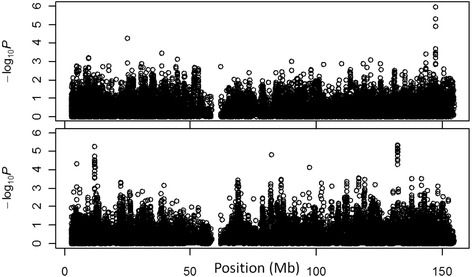
***Variance*** **and** ***standard*** **association tests point to different loci.** Manhattan plots are shown for the X chromosome for both the *variance* test (top) and a *standard* association test (bottom) for association with systolic blood pressure in the ARIC study.

We applied the three newly proposed tests to the ARIC data, as well as a standard association test. We included a total of 24313 X-linked SNPs and 7 quantitative traits, including total cholesterol (TC), low-density lipoprotein cholesterol (LDL-C), high-density lipoprotein cholesterol (HDL-C), triglyceride (TG), systolic blood pressure (SBP), diastolic blood pressure (DBP), and body mass index (BMI). Using a Bonferroni-corrected significance level for ChrX and one trait, we identified only one significant association for SBP using the *variance* test (Table 4). Interestingly, our results indicate that signals with this *variance* test are not in the same loci as those with a standard association test, in line with these two capturing very different types of signals (Figure 1). Specifically, the most significant locus associated with SBP according to the *variance* test is not detected by the *standard* association test, and vice versa. The most significant SNP in the novel locus discovered with the *variance* test is rs4427330 (*P* = 1.1 × 10^−6^; *P*_c_ = 0.027 following conservative Bonferroni correction for the number of X-linked SNPs tested). In contrast, no SNPs are chromosome-wide significant using the *standard* test. We found rs4427330 to also be nominally associated with DBP (*P* = 5.6 × 10^−4^). These results support the unique perspective added by tests for increased variance in heterozygous females in associating X-linked QTLs. Finally, in reported results of association with blood pressure from the Framingham Heart Study, rs4427330 has been nominally significant, though not reported since did not meet genome-wide significance [41]. Rs4427330 is located upstream of gene *AFF2* (also called *FMR2*), which might regulate splicing of *ATRX*, a gene that is associated with alpha-thalassemia (as a component of X-linked alpha-thalassemia mental retardation syndrome) [42]. The type of thalassemia observed in this disorder (Hb H thalassemia) can cause anemia and has been associated with hypertension [43].

**Table 4:** **P-values of four association tests between SNP rs4427330 and 7 quantitative traits in ARIC**

**Tests**	**TC**	**LDL**	**HDL**	**TG**	**SBP**	**DBP**	**BMI**
Standard	0.37	0.46	0.37	0.77	0.90	0.89	0.56
Variance	0.28	0.7	0.95	0.12	1.1 × 10^−6^	5.6 × 10^−4^	0.22
Weighted	0.77	0.62	0.37	0.62	0.45	0.59	0.096
Combined	0.54	0.72	0.82	0.27	2.9 × 10^−4^	0.016	0.071

**Figure 2:**
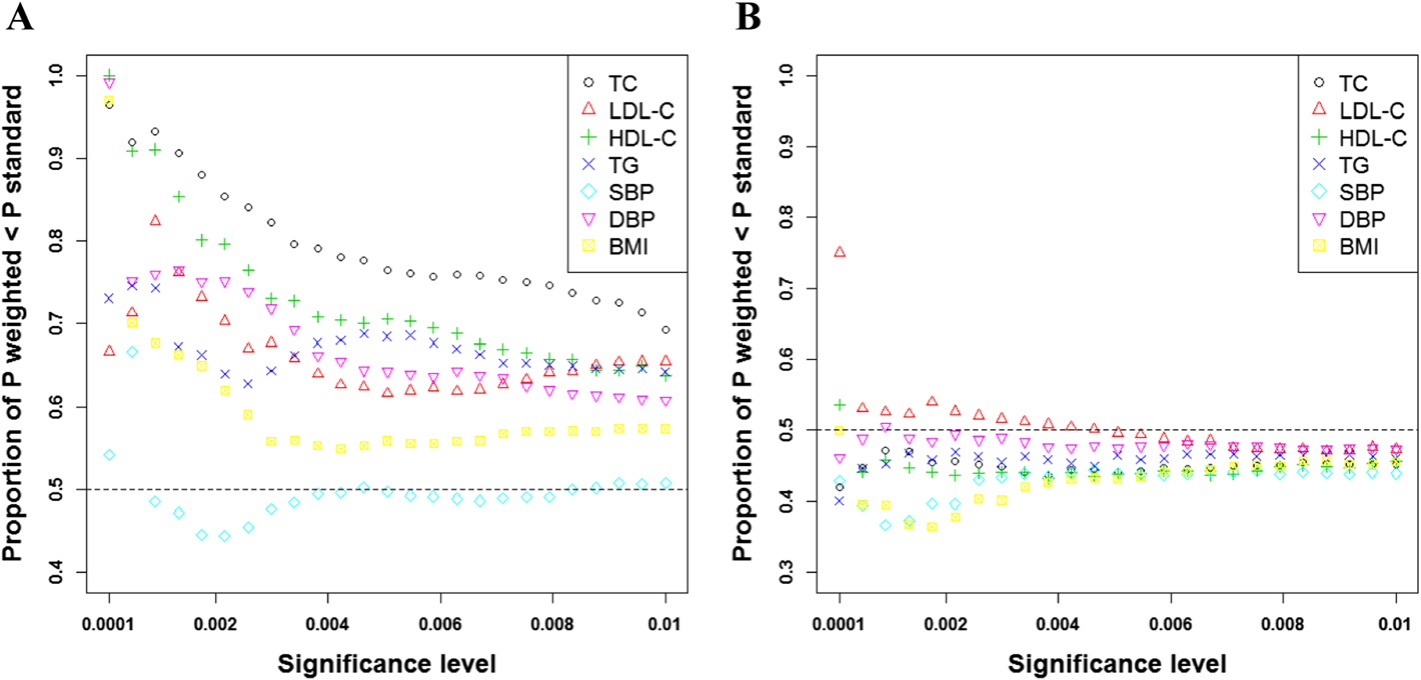
**Power improvement of weighted association test for the X chromosome (A) and the autosomes (B).** For all SNPs with p-value below a nominal significance level (x-axis) in either the *weighted* test or the *standard* association test, the figure presents the fraction that have a more significant p-value in the former. Colors denote different traits. Fraction greater than 0.5 (dotted horizontal line) and its increase with significance level both point to higher power of the weighted association test over the standard test for chromosome X **(A)**, while the trend is not obvious for the autosomes **(B)**.

Although no significant associations were identified using the other two variance-based tests, we further compared the power between the *weighted* and *standard* association tests using those empirical results. To reduce noises, only the top associated SNPs were included in the comparison with at least one of the two p-values (*weighted* and *standard* tests) smaller than a cutoff value. If the two tests are equally powerful, we expect the proportion of SNPs with a smaller p-value from the *weighted* test to be 50%. We observed that the *weighted* test was more powerful than the *standard* test for X-linked SNPs for six out of the seven traits across all the cutoff values in the ARIC data with the difference being larger for smaller or more stringent cutoff values (Figure 2A). In addition, the *weighted* test provided a smaller p-value than the *standard* test for all the seven traits on the top associated SNP from the standard test (Table 5). However, the same analysis applied to the autosomes failed to reveal the trend (Figure 2B), suggesting X-inactivation that is unique to chromosome X might have a larger effect on the variance heterogeneity than other factors that are associated with both chromosome X and autosomes. These empirical results from the ARIC study further support the existence of variance heterogeneity on chromosome X in real data and the potential of increasing power by incorporating it.

**Table 5:** **P-values of the standard and weighted association tests on the top associated SNP from the standard test for 7 quantitative traits in ARIC**

**Trait**	**Top SNP**	**Standard**	**Weighted**
TC	rs182215359	2.0 × 10^−5^	3.4 × 10^−6^
LDL	rs2257384	4.3 × 10^−7^	1.9 × 10^−6^
HDL	rs6530184	1.1 × 10^−4^	9.5 × 10^−5^
TG	rs5934418	7.3 × 10^−6^	2.4 × 10^−6^
SBP	rs5905825	1.2 × 10^−5^	1.2 × 10^−5^
DBP	rs7885152	2.8 × 10^−6^	2.6 × 10^−6^
BMI	rs1120140	5.7 × 10^−6^	3.8 × 10^−6^

The weighed test outperformed the standard test, even though this comparison favored the standard test by using SNPs that had the smallest p-value from the standard association test for each of the seven traits.

## Conclusions

In this study, we demonstrated a phenomenon of inflated phenotypic variation in females that are heterozygous for an X-linked QTL compared to females that are homozygous, which can be caused by random X-inactivation and other factors. Inspired by this, we proposed several tests for associating X-linked QTLs that are based on either directly testing for the inflated phenotypic variance or accounting for it as part of the testing for mean phenotypic effect. We have shown by simulations that the variance-based test captures different signals than the standard association test, thus can be used as a complementary test. After studying the power of these tests by simulations, we applied them to GWAS data from the ARIC study and identified an association between rs4427330 and systolic blood pressure that is not captured by standard association testing.

The newly proposed tests have similar or slightly better power than a standard association test in certain scenarios, but they capture unique signals using a different type of information based on variances, as demonstrated in simulations and the analysis of ARIC data. We therefore recommend using the variance-based tests as a complementary test to the standard mean-based test. While our simulations are restricted to a simplistic scenario of complete and random X-inactivation, these results point to the potential of a test of X-linked variance heterogeneity and for improvement in power of X-linked association testing when variance heterogeneity is involved. Interestingly, our results indicate that signals with this test are not in the same loci as those with a standard association test. The low correlation between the two statistics reinforces the fact that they each seek different features in the data.

Combined with our simulation studies that support a potential improvement in power when variance heterogeneity is involved, these results suggest that these tests merit further investigation. We will continue to develop such statistics and apply them to datasets that may reflect different types or levels of X-linked variance heterogeneity. For example, an ANOVA-like test for variance heterogeneity in general—rather than inflated variance in heterozygous females—can be similarly derived. We think this work will also pave the way to more sophisticated test statistics that combine the variance heterogeneity and tests of association of the means that further increase the power for detecting X-linked associations. Note that these variance-based tests are for quantitative traits, but can be potentially generalized for binary traits by making them quantitative so their variance can be considered, e.g. via liability threshold modeling [44]. We also hope this work will provide the incentive for the analysis and re-analysis of underutilized data for the X chromosome in many genome-wide association studies.
